# Supplementary material for: Patterns of Intron Gain and Loss in Fungi
Source: PLoS Biol. 2004 Nov 30;2(12):e422. doi: 10.1371/journal.pbio.0020422 (PMC532390; doi:10.1371/journal.pbio.0020422)
Supplement: Table S1 — Also available at http://genes.mit.edu/NielsenEtAl/. (4.3 MB ZIP). [file pbio.0020422.st001.zip › NielsenEtAl/html/68.html]

AN5599.1.NCU05985.1.MG01291.1.FG08766.1


```
 CLUSTAL W (1.82) Multiple Sequence Alignments - Introns Inserted


Sequence 1: NCU05985.1	829 aa
Sequence 2: MG01291.1	789 aa
Sequence 3: FG08766.1	1163 aa
Sequence 4: AN5599.1	749 aa
Alignment Length: 1230 aa
Number Identitical Residues: 314 aa
Alignment Score (without introns) 16454


MG01291.1 	MATN-------GPATKIG-------APMPDKDLEKKP------------------MELYP
NCU05985.1	MAEQSQQLNGHAPESHQHPNGNAETTPLLSTDLQQQHEGKDASAPPQSAKSPSASKELYP
FG08766.1 	MSKQAGTEDAAAPQSVTNPTSQN---------------------------------ELYP
AN5599.1  	MAKKP---------------------------------------------------YIPP
          	*: :.                                                    : *

MG01291.1 	MVSWKYDTLLWVMSIIADLFFREIHPRSSWKIPKHGPILFVAAPHANQ0FVDALVLQKTL
NCU05985.1	MSNWKYDTFLWTMSFLVDTFFREVHPRGSWKVPRSGPVLFVAAPHANQ0FVDALVLQRTL
FG08766.1 	MVNWKYDMFLYTVGNIVDMFFREVVPRGAWRVPQTGPVLFVAAPHANQ0FVDAIILQRTL
AN5599.1  	LIGWLYDLVLWTFSVLVDLFFREVHPRGSWKIPRRGPMIIVAAPHANQ0FVDSLILMRVL
          	: .* ** .*:... :.* ****: **.:*::*: **:::******** ***:::* :.*

MG01291.1 	KKEAGRRVSLLIAQKSIHG-FIGWASRQVGCVPVGRAQDAARPATGTIYLPNPIYDPTLI
NCU05985.1	RNEAKRRVSLLIAQKSVKG-FIGWASRQVGSVPVGRPQDSAKPAEGTIYLPDPIDDPTLI
FG08766.1 	RNEAKRRASLLIAQKSVHG-FIGWGSRQVGSVPVGRAQDAAKPATGTIYLPDPINDPTLI
AN5599.1  	RTEAHRRISFLIAEKSFRRKFIGLLARGIGTVPVARAMDMLKPGQGTVYLPDPVNQPTLL
          	:.** ** *:***:**.: .***  :* :* ***.*. *  :*. **:***:*: :***:

MG01291.1 	RGVGTKFDI-EAEVGGSLFLPSVKGQTGSHVEIAEIISPEELRIKRALKGKLPMEQLTGR
NCU05985.1	RGVGTNFEK-QAEVGGIIFLPSIKGQSGSNVEIVEIHGPEEIRIKRPVNSKLALYQLTGR
FG08766.1 	RGVGTKFGQGEGEVQGMLFLPSVKGQSGASVDILEIISPEEIRVKRPFKGKLALSQLTGR
AN5599.1  	RGVGTNFEAPGFEKDGTIALPTING-TSHSTSIAEIRGPEELILKKPFTHKDALFQLTGR
          	*****:*     *  * : **:::* :.  ..* ** .***: :*:... * .: *****

MG01291.1 	DDVDQDGIFKNKEVRSRKEGYQGTKYKLAPHIDQSKVYEAVFQRLRSGGCVGIFPEGGSH
NCU05985.1	DDIDEHGELQNKEVKGIKDGYQGTKFKIAPHIDQTKVYQAVFDRLKNGGCVGIFPEGGSH
FG08766.1 	DDIDNNGNFTIKEVKGPAPGFQGTKFKMAPHIDQTKVYDAVFSRLCNGGCVGIFPEGGSH
AN5599.1  	TDITDDGTFTGDVSDKDRADFKGSKFKAAPHVDQSAVYEAVFGRLLGGGCIGIFPEGGSH
          	 *: :.* :  .       .::*:*:* ***:**: **:*** ** .***:*********

MG01291.1 	DRTELLPLKP~GVAIMALGAMANYPDVGVKIVPVGMNYFHAHKFRSRAVVEFGNAIEVDP
NCU05985.1	DRSELLPLKA~GVAIMALGTLAEAPDCGLKIVPVGMNYFHPHKFRSRAVLEFGAPFEVPK
FG08766.1 	DRTELLPLKA~GVAIMALGTLAQDPDCGLKIVPVGMNYFHAHKFRSRAVVEFGAPFEIPR
AN5599.1  	DRPDLLPLKP1GVALMALGTLAENPDCGLKIVPCGMNYFHAHKFRSRAVIEFGTPLEVPK
          	**.:*****. ***:****::*: ** *:**** ******.********:*** .:*:  

MG01291.1 	ELVHMY-KNNQRREAIGTLLDTVHQGLSAVTVSTPDYDTLM~LIQAARRLYNPTGKKLPL
NCU05985.1	ELVEMYGIKDKRRAAISKLLDMVHQSLSAVTVSAPDYDTLM~VIQAARRLYNPAGKKLPL
FG08766.1 	HLVELY-CNKQRREAIGQVLDTVYQALNSVTVSAPDYDTLM~VIQAARRLYNPTGKKLPL
AN5599.1  	ELVEKY-KNGDRRGAVGPLLEMIYQSLVAVTVTSPDYETLM0VIQAARRLYNTKGKKLPL
          	.**. *  : .** *:. :*: ::*.* :***::***:*** :*********. ******

MG01291.1 	PVVVELNRRLAVGYERFKDDERVIKVRESVKKYNKNLRYLGIRDHQVQVTRMSWHRVVRI
NCU05985.1	PIVVELNRRLALGYDKYKDDPRIIELQKSVAEYNKQLRYLNIRDHQVQYAKLSWPKVVIT
FG08766.1 	PVVIELNRRLCMGYERYKNDERITSLTAAVKEYNSQLRYLNLKDHQVQYAKMNMVKVIFL
AN5599.1  	PMVVELNRRLVKGYSHFKDDPRIVDLKKSIADYNKQLRLLGIRDHQVEYAKFSFVKVIAT
          	*:*:******  **.::*:* *: .:  :: .**.:** *.::****: :::.  :*:  

MG01291.1 	FLYRILQLAVFFVATFPGLVLFGPVFIVAKVISIKKARSALAASTVKIQGRDVMATWKLL
NCU05985.1	FFYRVAKLLVLSAGVLPGLILFAPVFIATKRISHQKAKEALAGSTVKIQGKDVIATWKML
FG08766.1 	FIYRSIKLLLLFICTLPGLILFAPVFVATRIISRRKAKTALAGSTVKIRGRDVMATWKIL
AN5599.1  	LIYRLGKLALLTIGTLPGLLLFTPVFITTKLISNKKSKEALAASTVKLQGRDVMATWKLL
          	::**  :* ::   .:***:** ***:.:: ** :*:: ***.****::*:**:****:*

MG01291.1 	VSMAFAPIMYHVYCSLLAWKVARDGLWGFVPAWIVSLPWFVLVASIWLIGWPTFISITFA
NCU05985.1	VAMAVAPILYNLYTCLLAYMVGKHRLWGYVPEWVP-------IWLVYFFGWVFFPAITFA
FG08766.1 	VAIGLAPTLYHIYSAIVTFKVWQDRLWGRVPEGVP-------LWVIYFAMWPFMVGITFA
AN5599.1  	VALAFAPALYAFYTAAFTYWAYRNRINGYVPDFVP-------LWSFIPIGMVLFPTITFA
          	*::..** :* .* . .:: . :. : * **  :        :  .       :  ****

MG01291.1 	ALRFGEIGMDVFKSLRPLLLCMFPSSKYNMQKLQEQRAELSAEVTNLINTLGPEIFPDFA
NCU05985.1	ALRFGEVGMDILKSLRPLALCISPTSSYSLQSLKQRREELSAQVTHLINTLGPEMFPDFE
FG08766.1 	SLRFGEVGVDIFKSLRPLLLCLTPTSNYNIHRFRIRRAELSAQVTDVINTLGPEMFDDFE
AN5599.1  	ALRIGEIGMDIIKSLRPLLLSLNPSSANTLVKLRLRRAALAKQVTDTINTLGPELFPDFD
          	:**:**:*:*::****** *.: *:*  .:  :: :*  *: :**. *******:* ** 

MG01291.1 	QSRLIKASMDQHGAPLSPTHTRQPDSPTLKRRSDSELSFTGQASPGTSLHHPTSSNGIQR
NCU05985.1	HIRLVAADGSSR---IHADGTVSPTQP-FKRR-DSGVSSPPISTPGISTPGTESPHGLRR
FG08766.1 	KARLVPD--------LYKVEGGAATSPMSHRRRDSDQSSTGLEP--------ETPPALSR
AN5599.1  	AARIVTDP--------FRELNRQSANPEGEQRPPSVEIRR------------ASTENFDE
          	  *::                  . .*  .:*  *                  :.  : .

MG01291.1 	R-NTQSSRAIPRNESFSNIGKAGIFATRPPS----RNRSRTSSMGG------FN------
NCU05985.1	TGTTSSSRNIPRNESFSNIGQVPMFATRPPS----RSRSRSSSAGGGGGNVQFGGPLGGS
FG08766.1 	RSTTQSSRALPRNDSFSNIGHVGIFSTRPPSR--ARSRSRSSSSGGGFGSGGFP------
AN5599.1  	G--SPSQEPLPRNESFHNLANFGFFSTRPSSRNRSRSNSRSSSFGGLPGTSGQQ------
          	   : *.. :***:** *:.:  :*:***.* . :*..**:** **  ..          

MG01291.1 	-VQAFTALDSQEGFDEASRKIREAMRER-----------------------GEMRR---~
NCU05985.1	PVDKFPNLNTEEGFNEASKKIREAMRER-----------------------GEMRR---~
FG08766.1 	-LSGFTTLDSSGGFDEATRKIREAMKHRRHKTDQERTDGDEEDDDSEEEGYDEARKKNA2
AN5599.1  	-LKPLSQLTTKGGFDEVSSKIRDAMRER-----------------------GVQRR---~
          	 :. :. * :. **:*.: ***:**:.*                       .  *:    

MG01291.1 	------------~-----RRS~E-------------------------------------
NCU05985.1	------------~-----RKS~Q-------------------------------------
FG08766.1 	TWIDTSNEEWLC1SVIGVRRG1QPHNPSSSTTAATPSWLPRPPAKQRMALSFKNLVSSVL
AN5599.1  	------------~-----RRS~E-------------------------------------
          	                  *:. :                                     

MG01291.1 	----------------------------------------ARGMMIGGS-----------
NCU05985.1	----------------------------------------GKVYTFSEQ-----------
FG08766.1 	GSGAAKDDKPPSASELFVKTDPKVDGEECLHDCDNCSARYPRGFKIEEEDVLYGLVAAWS
AN5599.1  	------------------------------------------------------------
          	                                                            

MG01291.1 	----------------EDEEEDHDDENS-----SDG~KKNA-------------------
NCU05985.1	----------------SVDEEVEDEVSTGSSVDEDG~KKHL-------------------
FG08766.1 	THVLVGTGKTDWVRDVEEEEGSVMQAFSKAVEPSNG0KLKLSASNMPTPHDTDDYSEPTT
AN5599.1  	----------------DNSSWDMASSGPGTPHSEDN~RKDI-------------------
          	                . ..    .  . :   .:. : .                    

MG01291.1 	------------------------------------------------------------
NCU05985.1	------------------------------------------------------------
FG08766.1 	LLLLPAFKLLRNVHPLSVPQIITEVINQAPTNTSPLTPTPLPASLPSPDPAAGIPDVSLA
AN5599.1  	------------------------------------------------------------
          	                                                            

MG01291.1 	------------------------------------------------------------
NCU05985.1	------------------------------------------------------------
FG08766.1 	NCPHNAVILMCSHRTRDVRCAQSAPVLRKEFERQLRPLGLYRDLHDERPGGVGIYYISHV
AN5599.1  	------------------------------------------------------------
          	                                                            

MG01291.1 	------------------------------------------------------------
NCU05985.1	------------------------------------------------------------
FG08766.1 	GGHKYSANVMIYRRPNAFGLDDTTEQQNGTSNGEKNGDSVNDSGEGSVGAAQGIWLARVM
AN5599.1  	------------------------------------------------------------
          	                                                            

MG01291.1 	-------------------------------------
NCU05985.1	-------------------------------------
FG08766.1 	PEDCENLIRYTVLRGKVVKPERQLRGGFDRGRGVMSW
AN5599.1  	-------------------------------------
          	
```
